# The Role of Microbial Metabolites in the Progression of Neurodegenerative Diseases—Therapeutic Approaches: A Comprehensive Review

**DOI:** 10.3390/ijms251810041

**Published:** 2024-09-18

**Authors:** Jorge Missiego-Beltrán, Ana Isabel Beltrán-Velasco

**Affiliations:** NBC Group, Psychology Department, School of Life and Nature Sciences, Nebrija University, 28015 Madrid, Spain; jmissiegob@alumnos.nebrija.es

**Keywords:** gut microbiota, microbial metabolites, neurodegenerative diseases, short-chain fatty acids, prebiotics, probiotics, fecal microbiota transplantation

## Abstract

The objective of this review is to provide a comprehensive examination of the role of microbial metabolites in the progression of neurodegenerative diseases, as well as to investigate potential therapeutic interventions targeting the microbiota. A comprehensive literature search was conducted across the following databases: PubMed, Scopus, Web of Science, ScienceDirect, and Wiley. Key terms related to the gut microbiota, microbial metabolites, neurodegenerative diseases, and specific metabolic products were used. The review included both preclinical and clinical research articles published between 2000 and 2024. Short-chain fatty acids have been demonstrated to play a crucial role in modulating neuroinflammation, preserving the integrity of the blood–brain barrier, and influencing neuronal plasticity and protection. Furthermore, amino acids and their derivatives have been demonstrated to exert a significant influence on CNS function. These microbial metabolites impact CNS health by regulating intestinal permeability, modulating immune responses, and directly influencing neuroinflammation and oxidative stress, which are integral to neurodegenerative diseases. Therapeutic strategies, including prebiotics, probiotics, dietary modifications, and fecal microbiota transplantation have confirmed the potential to restore microbial balance and enhance the production of neuroprotective metabolites. Furthermore, novel drug developments based on microbial metabolites present promising therapeutic avenues. The gut microbiota and its metabolites represent a promising field of research with the potential to advance our understanding of and develop treatments for neurodegenerative diseases.

## 1. Introduction

Neurodegenerative diseases represent a significant global health burden, accounting for a considerable proportion of disability and mortality. Alzheimer’s Disease (AD), the most prevalent form of dementia, affects over 50 million individuals globally. Projections indicate that this figure will increase threefold by 2050, largely as a result of population aging. Parkinson’s Disease (PD) is the second most prevalent neurodegenerative disease, affecting more than 10 million individuals worldwide, with an incidence that is also on the rise. In contrast, Multiple Sclerosis (MS), although less prevalent, affects approximately 2.8 million individuals globally, with a notable variation in geographical distribution, being more prevalent in temperate regions. These diseases not only represent a substantial burden on healthcare systems but also significantly impact the quality of life of patients and their families due to the progressive loss of neurological function that characterizes these conditions [1,2,3].

From a clinical standpoint, the defining characteristic of these pathological conditions is a gradual and progressive deterioration of the function of the neuronal cells in the brain. This ultimately manifests as a constellation of debilitating clinical symptoms. Among the most prevalent manifestations are the progressive diminution of cognitive, motor, and sensory functions, which vary according to the specific type of disorder [4,5]. In AD, the most prevalent symptoms are a gradual decline in memory, disorientation in time and space, difficulties in abstract thinking and reasoning, and alterations in behavior and personality [6,7]. PD is characterized by the presence of motor symptoms, including tremors at rest, muscle rigidity, bradykinesia, and problems with balance and coordination. These symptoms are attributed to the degeneration of dopaminergic neurons in the substantia nigra of the brain [8]. MS presents a spectrum of symptoms that may include muscle weakness, coordination and balance problems, blurred vision, numbness or tingling sensations, and difficulties in bladder control. These symptoms are the result of the destruction of the myelin that covers the nerve fibers in the central nervous system (CNS) [9].

From a biochemical standpoint, neurodegenerative diseases are defined by intricate pathological cascades. These include the aggregation of misfolded proteins, mitochondrial dysfunction, oxidative stress, neuroinflammation, and neuronal death. Despite significant strides in elucidating these mechanisms, current treatment strategies remain inadequate. They are predominantly palliative in nature and lack the capacity to halt or reverse disease progression [10,11,12].

In this context, the study of the microbiota–gut–brain axis has emerged as a new frontier in neurodegenerative disease research. This axis comprises a complex network of bidirectional communication between the gastrointestinal tract and the CNS, in which the intestinal microbiota plays a pivotal role. The intestinal microbiota, comprising millions of microorganisms, including bacteria, viruses, fungi, and protozoa, is involved in a multitude of physiological functions, ranging from nutrient digestion to immune system regulation. Moreover, it has been established that the microbiota exerts a profound influence on mental and neurological health, affecting behavior, mood, and cognitive function [13,14,15].

One of the principal mechanisms through which the gut microbiota exerts an influence on the CNS is via the production of metabolites. These bioactive compounds, which include short-chain fatty acids (SCFAs), neurotransmitters such as gamma-aminobutyric acid (GABA) and serotonin, secondary bile acids, and various compounds derived from the amino acid metabolism, can directly influence brain function. Microbial metabolites can cross the blood–brain barrier (BBB), act on neuronal receptors, modulate neuroglial inflammation, and alter intestinal barrier permeability, among other effects [16,17].

Recent research has provided compelling evidence that gut dysbiosis, defined as an imbalance in the composition and function of the microbiota, contributes to alterations in the production and profile of metabolites, which may play a role in the pathogenesis and progression of neurodegenerative diseases [13,18]. For example, in AD, it has been observed that certain microbiota-derived metabolites can promote the formation of amyloid plaques and exacerbate neuroinflammation [19]. In PD, the altered production of SCFAs and tryptophan metabolites can influence the death of dopaminergic neurons and the appearance of motor and neuropsychiatric symptoms [20]. Likewise, in MS, dysbiosis can modulate the immune response through changes in microbial metabolites, affecting demyelination and chronic inflammation [21].

Despite mounting evidence indicating the pivotal role of microbial metabolites in neurodegenerative disorders, the precise manner by which distinct metabolites affect the various phases of the disease remains uncertain. Additionally, the potential of modulating the gut microbiota through dietary interventions, probiotics, prebiotics, or fecal microbiota transplantation (FMT) as an efficacious therapeutic approach remains to be elucidated. Furthermore, existing studies have demonstrated considerable inter-individual variability, suggesting that the microbial composition and its impact on the CNS may be highly personalized. Given the early stages of research into microbial metabolites in neurodegenerative diseases, the potential for new therapeutic strategies is significant. However, a more comprehensive and detailed understanding of the underlying molecular mechanisms and methods for safely and effectively manipulating them in a clinical context is essential. Advancements in this field of research have the potential to pave the way for the development of novel personalized therapeutic modalities that not only alleviate the associated clinical symptoms but also alter the progression of neurodegenerative diseases.

## 2. Objectives and Methodology

The principal aim of this review is to examine the part played by microbial metabolites in the development of neurodegenerative disorders, with a particular emphasis on the molecular mechanisms through which they exert an influence on neuroinflammation, neuroprotection, and synaptic dysfunction. By undertaking a comprehensive analysis of the available literature, we seek to present a comprehensive overview of the manner in which these metabolites may act as key modulators in the context of these pathologies, and to address potential therapeutic interventions based on the modulation of the intestinal microbiota.

The specific objectives of this review are as follows:

1. To describe the composition and metabolic functions of the intestinal microbiota.
2. To analyze the main microbial metabolites and their mechanisms of action in the CNS.
3. To review the scientific evidence linking these metabolites to the pathogenesis of specific neurodegenerative diseases.
4. To examine therapeutic interventions targeting the intestinal microbiota and its metabolites as potential strategies to prevent or mitigate the progression of neurodegenerative diseases.

A comprehensive literature search was conducted in the following scientific databases: PubMed, Scopus, Web of Science, ScienceDirect, and Wiley. To optimize the retrieval of pertinent articles, specific keywords were combined with Boolean operators. The primary search terms included “gut microbiota”, “microbial metabolites”, “neurodegenerative diseases”, “short-chain fatty acids”, “neuroinflammation”, “blood-brain barrier”, “Alzheimer’s”, “Parkinson’s”, and “Multiple Sclerosis”. The search was limited to studies published in English between 2010 and 2024 to ensure the inclusion of the most recent and relevant research.

The inclusion criteria were as follows: (i) original preclinical or clinical research studies examining the relationship between gut microbiota, its metabolites, and neurodegenerative diseases; (ii) systematic reviews and meta-analyses providing a comprehensive summary of the existing literature; (iii) studies analyzing specific mechanisms of action of microbial metabolites in the CNS.

The following articles were excluded: (i) those which did not provide relevant data on the interaction between microbiota and neurodegenerative diseases; (ii) those which were limited to in vitro studies without a clear link to in vivo models of neurodegeneration; (iii) those which were case reports, editorials, or comments without an empirical basis.

The study selection process was conducted in two distinct phases. Initially, the titles and abstracts of the initially identified articles were reviewed to ascertain those that potentially met the pre-established inclusion criteria. The articles deemed eligible following this initial review were then subjected to a comprehensive second full-text examination. The final selection of studies was based on the following three fundamental criteria: the relevance of the content, the methodological quality, and the contribution to the advancement of our understanding of the role of the microbiota and its metabolites in neurodegenerative diseases.

The data extracted from the selected studies were synthesized in a qualitative manner. The review was organized into several thematic sections to address different aspects of the microbiota–metabolites–CNS interaction, including the production of microbial metabolites, their influence on neuroinflammation and the integrity of biological barriers, and their role in specific neurodegenerative diseases. Additionally, the review included a critical analysis of the methodological limitations of the studies and the variability in the results reported.

## 3. Gut Microbiota: Composition and Functions

The gut microbiota represents a complex and diverse ecosystem comprising millions of microorganisms. This microbial community exerts an essential influence on a range of vital physiological functions, which are critical to the health and homeostasis of the organism. Over the last few years, knowledge about the intestinal microbiota has advanced significantly as a result of the application of omics techniques, which have demonstrated the microbiota’s influence on processes extending from digestion to the modulation of the immune system and communication with the CNS [13,22].

### 3.1. The Diversity and Composition of the Gut Microbiota

The human gut microbiota is distinguished by remarkable diversity, with an estimated 500 to 1000 distinct bacterial species present in a typical individual. This diversity is vital for maintaining the functional equilibrium of this ecosystem and exhibits considerable variation between individuals, influenced by factors such as diet, age, environment, and the use of medications, particularly antibiotics [23].

Two of the most prevalent bacterial phyla in the human gut microbiota are *Bacillota* (formerly known as *Firmicutes*) and *Bacteroidetes*, collectively representing the majority of the bacterial community. *Bacillota* encompasses genera such as *Lactobacillus*, *Clostridium*, *Faecalibacterium*, and *Ruminococcus*. These bacteria are of particular significance in the fermentation of complex carbohydrates into SCFAs, which play pivotal roles in regulating the body’s metabolism and immune response [24,25].

Conversely, the *Bacteroidetes* phylum is predominantly represented by genera such as *Bacteroides* and *Prevotella*, which also play a pivotal role in the degradation of complex dietary polysaccharides. These processes contribute to the production of SCFAs, which are fundamental not only for intestinal health but also for communication with the CNS through the microbiota–gut–brain axis [26].

Additionally, the gut microbiota comprises *Actinobacteria* and *Proteobacteria*, though in lesser proportions than the previously mentioned dominant phyla. Bifidobacterium, a genus within the *Actinobacteria* phylum, is renowned for its capacity to ferment dietary fibers, yielding beneficial metabolites that can modulate the immune system and contribute to gut health. Conversely, bacteria belonging to the *Proteobacteria* phylum, such as *Escherichia coli*, are less prevalent in a healthy gut. However, an increase in their proportion has been linked to states of inflammation and dysbiosis, underscoring their pivotal role in maintaining microbial balance [27].

The diversity and stability of the gut microbiota play a pivotal role in the maintenance of host health and well-being. The loss of this diversity, otherwise known as dysbiosis, has been linked to a range of pathological conditions, including those related to metabolism, inflammation, and neurodegeneration. Consequently, there is a pressing need to comprehend not only the dynamics of this microbial diversity but also the influence it exerts on human health. This knowledge is vital to the development of interventions capable of restoring and maintaining equilibrium within the gut microbiome [28].

### 3.2. Key Metabolic Functions of the Gut Microbiota

The gut microbiota plays a multifaceted role in the digestive and absorptive processes, as well as in the production of a vast array of bioactive metabolites that exert systemic effects [29]. Among the most significant metabolites are SCFAs, which encompass acetate, propionate, and butyrate. These SCFAs are the end products of the bacterial fermentation of non-digestible dietary fibers and possess a multitude of beneficial functions. They serve as energy sources for colonocytes, modulate inflammation through the regulation of cytokine production, and maintain the integrity of the intestinal barrier [30].

In addition to SCFAs, the gut microbiota is involved in the synthesis of several essential vitamins, including B vitamins (B7, B9, and B12) and vitamin K. These vitamins play a crucial role in a number of biological functions, such as neurotransmitter synthesis, blood cell formation, and blood coagulation regulation [31]. Deficiency in these vitamins, frequently resulting from alterations in the microbiota, can have considerable adverse effects on the nervous system and contribute to the progression of neurodegenerative diseases. Other metabolites, such as secondary bile acids, which result from the modification of primary bile acids synthesized by the liver, are also produced. These metabolites are not only involved in lipid digestion, but also act as signaling molecules that can influence inflammation and energy homeostasis [32].

Another significant group of metabolites produced by the microbiota are amino acid derivatives, including tryptophan. The metabolism of tryptophan by the intestinal microbiota can result in the production of indoles, indole-3-carboxylic acids, and quinolinic acids, which have notable implications in the modulation of the immune system and in neuroprotection [19]. Moreover, these metabolites can traverse the BBB and interact with neuronal receptors, directly influencing brain function and neurogenesis [19,33].

Other relevant metabolic function is the modification of endogenous and exogenous compounds, including the biotransformation of drugs and the metabolization of toxins and hormones. The intestinal microbiota can alter the bioavailability and activity of various drugs, affecting their efficacy and toxicity [34,35]. Furthermore, the metabolism of hormones such as estrogens and catecholamines can influence the inflammatory state and oxidative stress, both of which are relevant factors in neurodegeneration [36].

Additionally, the gut microbiota is capable of synthesizing neurotransmitters such as GABA and serotonin, which are vital for communication between the gut and the brain. These neurotransmitters can influence mood, behavior, and cognition, emphasizing the significance of the microbiota in the gut–brain axis [37].

### 3.3. Interaction between Gut Microbiota and the Immune System

The gut microbiota plays a pivotal role in the development, maturation, and function of the immune system, particularly within the gastrointestinal tract, where the majority of the body’s immune system resides. The relationship between the microbiota and the immune system is highly dynamic and bidirectional, with the microbiota exerting an influence over immune function and immune responses concurrently shaping the composition and function of the microbiota [38].

One of the mechanisms through which the microbiota modulates the immune system is through the production of SCFAs, including butyrate, propionate, and acetate. These SCFAs exert an anti-inflammatory effect by promoting the differentiation of regulatory T cells (Tregs) in the intestine [39]. Treg cells represent a specialized subgroup of T cells that play a critical role in maintaining immune homeostasis and preventing autoimmune diseases. They originate in the thymus and are distinguished by their expression of the CD4 marker and the FoxP3 transcription factor, which are fundamental for their development and function. There are two main types of Treg cells: natural Treg cells (nTregs) and induced Treg cells (iTregs) [40,41].

Natural regulatory T cells (nTregs) develop in the thymus and are specialized in regulating autoimmune responses. These cells are capable of suppressing the activation of effector T cells that could attack the body’s own tissues, thereby ensuring tolerance towards self-antigens and preventing the development of autoimmune diseases [42]. Induced regulatory T cells (iTregs) are generated in peripheral tissues under the influence of environmental factors and microenvironmental signals. These cells develop from naïve T cells in response to specific stimuli, such as the presence of antigens or the influence of particular cytokines. Their function is to maintain tolerance towards non-self-antigens and control inappropriate immune responses in peripheral tissues [43].

Treg cells perform their regulatory function through a number of different mechanisms. One of the principal mechanisms by which Treg cells exert their regulatory function is through the secretion of immunosuppressive cytokines, including interleukin-10 (IL-10) and transforming growth factor beta (TGF-β). These cytokines inhibit the activation and proliferation of other immune cells, such as effector T cells and B cells. Additionally, Treg cells have the capacity to induce apoptosis in activated immune cells and modify the function of antigen-presenting cells, thereby contributing to the reduction in inflammation and the prevention of autoimmune responses [44].

In addition to SCFAs, the gut microbiota also exerts influence over the production of other immunomodulatory molecules, including cytokines and chemokines. These molecules serve as pivotal mediators of communication between immune cells, thereby facilitating the orchestration of immune responses [45,46]. To illustrate, the production of proinflammatory cytokines, including interleukin-1β (IL-1β), tumor necrosis factor alpha (TNF-α), and interleukin-6 (IL-6), can be influenced by the microbiota. An increase in these cytokines has been linked to neuroinflammation in AD and PD, where the chronic activation of microglia, the resident immune cells of the CNS, has been observed. Activated microglia not only perpetuate inflammation in the brain but also contribute to neuronal death, exacerbating the pathology of neurodegenerative diseases [47].

One of the principal mechanisms through which the gut microbiota can influence brain inflammation is via the production of tryptophan-derived metabolites. Tryptophan is an essential amino acid that, in addition to its role in serotonin synthesis, is metabolized in the gut to produce a variety of bioactive compounds [48]. Among these metabolites, quinolinic acid is of particular interest due to its capacity to traverse the BBB and exert effects on the CNS. Quinolinic acid is a metabolite of the tryptophan cycle that functions as an agonist of glutamate receptors, particularly the N-methyl-D-aspartate (NMDA) receptor. This interaction has the potential to induce neuronal excitotoxicity and contribute to neuroinflammatory processes [49]. Elevated levels of quinolinic acid have been linked to heightened neuroinflammation and neuronal damage in numerous neurodegenerative disorders. In the context of MS, there is compelling evidence that this metabolite plays a pivotal role in the disease’s pathogenesis. The dysbiosis observed alters the production of this metabolite, leading to the aberrant activation of autoreactive immune cells, such as T and B cells, which migrate to the CNS through the BBB. Once in the CNS, these autoreactive cells can trigger a chronic inflammatory response that damages myelin and neurons, contributing to the clinical symptoms of the disease [50].

### 3.4. Gut Microbiota and the Integrity of the Intestinal Barrier

The intestinal barrier, constituted by a layer of epithelial cells interconnected by tight junctions, functions as a physical and functional barrier that separates the luminal contents of the intestine from the rest of the body. This barrier is vital for maintaining homeostasis, enabling the selective absorption of nutrients while preventing the translocation of pathogens and toxins. The intestinal microbiota plays a pivotal role in maintaining the integrity of this barrier [51].

SCFAs, particularly butyrate, are essential for maintaining the health of intestinal epithelial cells. They provide a source of energy and promote the expression of proteins that reinforce tight junctions, which are vital for preventing intestinal permeability. This condition occurs when the intestinal barrier is compromised, allowing bacteria, toxins, and microbial products to enter the bloodstream. This phenomenon can trigger a systemic immune response and chronic inflammation, which are risk factors for the development of neurodegenerative diseases [52].

In neurodegenerative diseases, an increase in intestinal permeability has been observed to be associated with an increase in the translocation of lipopolysaccharide (LPS), a component of the cell wall of Gram-negative bacteria. LPS is a potent activator of the immune system and can trigger a systemic inflammatory response affecting the CNS [53]. It has been demonstrated that LPS can cross the BBB, activate microglia, and promote the release of proinflammatory cytokines, which exacerbates neuroinflammation and contributes to disease progression [54]. In AD, it has been postulated that intestinal dysbiosis and increased intestinal permeability may be related to the accumulation of beta-amyloid plaques, a characteristic pathological finding of the disease [55,56].

Moreover, the gut microbiota plays a pivotal role in the modification and production of secondary metabolites, including modified bile acids, which exert a profound influence on intestinal and systemic health. These secondary metabolites are the result of the microbial transformation of primary bile acids, which are synthesized in the liver and released into bile [57]. Primary bile acids, such as cholic acid and chenodeoxycholic acid, undergo modification by the gut microbiota in the colon, resulting in the formation of secondary bile acids, including deoxycholic acid and lithocholic acid. Secondary bile acids possess the capacity to modulate inflammatory processes and local immune responses within the intestinal mucosa [58]. This is achieved through their interaction with membrane receptors on intestinal epithelial cells and on immune cells present in the mucosa. These acids can activate nuclear receptors, including peroxisome proliferator-activated receptors (PPARs) and bile acid receptors (TGR5). The activation of these receptors can influence a range of cellular processes, such as the proliferation, differentiation, and apoptosis of intestinal epithelial cells [59].

The modulation of the local immune response by secondary bile acids includes the regulation of the production of proinflammatory and anti-inflammatory cytokines in the intestinal mucosa. Alterations in this production may have a direct impact on systemic inflammation and, consequently, on CNS health [60,61]. One example is the increased production of proinflammatory cytokines, which may contribute to a chronic inflammatory state that extends to the brain, negatively affecting neuronal function and accelerating the progression of neurodegenerative diseases such as AD and PD [62].

**Figure 1:**
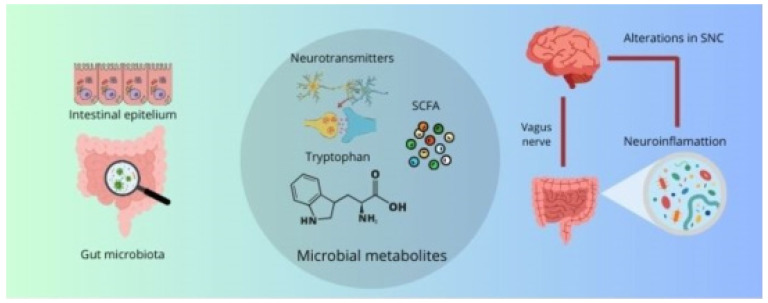
The gut microbiota produces metabolites that can exert effects on the CNS through bidirectional communication of the gut–brain axis, leading to neuroinflammation and CNS alterations.

The relationship between the gut microbiota, the immune system, and the intestinal barrier represents a fundamental component in the pathophysiology of neurodegenerative diseases. Intestinal dysbiosis, resulting in altered metabolite production, increased intestinal permeability, and dysfunctional modulation of the immune system, can create a proinflammatory environment in both the gut and the brain, contributing to the progression of neurodegenerative diseases. This comprehensive approach on the role of the microbiota and its interactions with the immune system and the intestinal barrier underscores the importance of considering intestinal health as a critical factor in the prevention and treatment of these pathologies (Figure 1).

## 4. Microbial Metabolites: Characteristics and Action Mechanisms

Microbiota metabolites are chemical compounds produced by gut bacteria during the digestion and metabolism of nutrients. The gut microbiota produces a wide variety of metabolites that exert local and systemic effects, modulating crucial health functions, including immune system regulation, inflammation, and neuronal function. These metabolites, which include SCFAs, amino acid derivatives, phenolic compounds, and other microbial products, play a fundamental role in gut–brain communication

### 4.1. Short Chain Fatty Acids (SCFAs)

SCFAs, including butyrate, propionate, and acetate, are produced through the fermentation of complex carbohydrates by the gut microbiota. Dietary fibers, including oligosaccharides, pectins, and cellulose, reach the colon, where they are fermented by specific gut bacteria. This fermentation process converts carbohydrates into SCFAs, gases, and other metabolites. The production of SCFAs varies depending on the composition of the microbiota and the diet of the individual, with different bacterial species contributing to the synthesis of these metabolites [63].

One of the most significant mechanisms of action of SCFAs is their capacity to serve as an energy source for colonocytes, the cells that line the colon. In particular, butyrate is essential for maintaining the health of these cells, promoting the integrity of the intestinal barrier and preventing the translocation of pathogens and toxins into the bloodstream [64]. SCFAs possess notable anti-inflammatory characteristics. At the molecular level, these fatty acids can inhibit the activity of histone deacetylase (HDAC), an enzyme that regulates gene expression. By inhibiting HDACs, SCFAs facilitate the expression of genes encoding proteins with anti-inflammatory effects, including anti-inflammatory cytokines and tight junction proteins in intestinal epithelial cells [65].

Moreover, butyrate has been demonstrated to facilitate the differentiation of Tregs within the gut. Its function is to suppress the activation of effector T cells and the production of anti-inflammatory cytokines [66]. A reduction in butyrate levels may therefore result in a decline in the Treg cell population and an intensification of systemic inflammation, which could, in turn, exacerbate the progression of neurodegenerative diseases. Acetate and propionate also exert significant effects on human health. Acetate, the most prevalent SCFA, serves as an energy source for a range of tissues and can influence appetite regulation and the lipid metabolism. Propionate, meanwhile, plays a role in cholesterol regulation and in the modulation of gluconeogenesis in the liver [67,68].

Conversely, SCFAs may impact neurotransmission within the brain by modulating the expression and functionality of neurotransmitters and their receptors [69]. Of the SCFAs, butyrate has attracted particular attention due to its marked effects on neuronal function. Butyrate has been demonstrated to modulate the activity of numerous neurotransmitters, including glutamate and GABA. Glutamate is the primary excitatory neurotransmitter in the brain and plays a role in synaptic plasticity and memory [70]. Butyrate may influence the glutamatergic system by regulating the expression of glutamate receptors and neurotransmitter release, thereby affecting neuronal excitability and synaptic signaling. Additionally, butyrate may also affect GABA-mediated inhibitory neurotransmission, which is crucial for maintaining the balance between excitation and inhibition in the brain [71].

Inhibition of HDACs by butyrate facilitates histone acetylation, resulting in a more relaxed chromatin structure and increased accessibility of genes for transcription, including those encoding BDNF. This increase in BDNF expression is of great consequence for neurogenesis, a process entailing the generation of new neurons from neural stem cells. Synaptic plasticity is also favored by elevated levels of BDNF, which may translate into enhanced learning and memory capacity, as well as a potential therapeutic avenue for mitigating the deterioration associated with neurodegenerative pathologies [72].

### 4.2. Amino Acids and Their Derivatives

Amino acids and their metabolites play a pivotal role in the communication between the intestinal microbiota and the CNS. Tryptophan and its derivatives, such as kynurenic acid, are of particular significance due to their capacity to modulate inflammation and neuronal excitotoxicity [73].

Tryptophan is metabolized by the gut microbiota into several bioactive compounds, including kynurenic acid, which has neuroactive properties. This metabolite functions as an antagonist of the NMDA receptor, a crucial receptor in glutamatergic neurotransmission and in the regulation of excitotoxicity. Excitotoxicity, resulting from the excessive activation of the NMDA receptor, represents a central pathological process in various neurodegenerative diseases [74]. By inhibiting the excessive activation of these receptors, kynurenic acid may exert a neuroprotective effect, reducing neuronal damage associated with diseases such as amyotrophic lateral sclerosis (ALS) and Huntington’s Disease (HD) [75].

GABA is synthesized by both the intestinal microbiota and the CNS. The equilibrium between GABA and glutamate is crucial for optimal brain function. The microbial production of GABA and its modulation via the gut–brain axis can impact neurological disorders characterized by an imbalance between excitation and inhibition, such as epilepsy and schizophrenia. Additionally, alterations in the intestinal microbiome, which influence the synthesis of GABA and other neurotransmitters, have been associated with mood and behavioral changes in neurodegenerative diseases, including depression and anxiety, which frequently accompany these pathologies [76].

Other amino acids, including phenylalanine and tyrosine, are also metabolized by the gut microbiota into compounds that exert neuromodulatory effects. These amino acids serve as precursors to key neurotransmitters such as dopamine and norepinephrine, which are involved in regulating mood, motivation, and cognitive function. Alterations in the production and metabolism of these amino acids and their derivatives may contribute to the neuropsychiatric symptoms observed in neurodegenerative diseases, as well as motor and cognitive dysfunction [77].

### 4.3. Polyphenols and Their Derived Metabolites

Polyphenols are natural compounds that are widely distributed in plants. They are known for their antioxidant and anti-inflammatory properties, as well as their beneficial effects on human health. Polyphenols are found in a wide variety of foods, including fruits, vegetables, tea, coffee, wine, and cocoa. However, due to their complex chemical structure and high molecular weight, the majority of polyphenols are not directly absorbed in the small intestine. Instead, they reach the large intestine, where they undergo a process of metabolization by the intestinal microbiota, resulting in a series of simpler and bioactive metabolites that can be absorbed by the body [78,79].

The gut microbiota plays a fundamental role in the transformation of polyphenols into active metabolites. Through hydrolysis, reduction, and demethylation processes, bacteria in the intestine break down polyphenols into smaller compounds with a higher absorption capacity, including phenolic acids, simple flavonoids, and other derived metabolites. For example, catechins, which are present in green tea, are metabolized into valeric acid and phenolic acids [80]. Similarly, anthocyanins, which are derived from red fruits, are transformed into phenolic acids that exhibit potent biological properties. Once metabolized, these compounds can be absorbed by the epithelial cells of the colon and enter the systemic circulation, where they exert their biological effects. Some of these metabolites are rapidly eliminated in urine, while others can accumulate in tissues and organs, modulating various physiological functions [81].

With regard to neuroprotective effects, polyphenol-derived metabolites are capable of crossing the BBB and exerting direct actions within the CNS. In the brain, these compounds have been demonstrated to inhibit the production of proinflammatory cytokines and reduce the activation of microglia, which are immune cells of the CNS that play a crucial role in the inflammatory response [82]. The inhibition of microglia can prevent or mitigate neuronal damage associated with chronic neuroinflammatory processes. Furthermore, polyphenols and their metabolites have potent antioxidant properties, neutralizing reactive oxygen species (ROS) and protecting neuronal cells from oxidative stress, which is a key factor in neurodegeneration. For example, gallic acid, a common metabolite of polyphenols, has demonstrated its ability to reduce oxidative damage in experimental models of neurodegeneration [83].

Additionally, they impact neuronal signaling and synaptic plasticity. Certain polyphenol metabolites have been demonstrated to elevate levels of neurotrophic factors, such as BDNF, which is crucial for neuron survival, neurogenesis, and synaptic plasticity. Enhancing synaptic plasticity is especially pertinent in the context of neurodegenerative diseases, where impaired synaptic function contributes to memory loss and cognitive decline [84,85]. Moreover, polyphenols may impact neurotransmission by modulating the release and reception of pivotal neurotransmitters, including dopamine, serotonin, and GABA. This effect is partially attributed to the capacity of polyphenols to interact with receptors for these neurotransmitters, thereby regulating their levels in distinct regions of the brain. This may subsequently influence mood, anxiety, memory, and other cognitive functions [86,87].

Flavonoids represent a significant subset of polyphenols, constituting one of the largest and most diverse groups within this class of compounds. Among flavonoids, quercetin is particularly noteworthy for its prevalence in foods such as apples, onions, grapes, and broccoli. It is widely recognized for its potent antioxidant and anti-inflammatory activity. Catechins, another subgroup of flavonoids, are found in large quantities in green tea, cocoa, and grapes [88]. Of particular note is epigallocatechin gallate (EGCG), which has been demonstrated to possess antioxidant and cardioprotective effects. Anthocyanins, which are responsible for the intense colors observed in fruits and vegetables such as berries, grapes, cherries, and eggplants, have also been shown to possess antioxidant properties and to improve cardiovascular and cognitive health [89,90].

In addition to flavonoids, phenolic acids represent another class of significant polyphenols. Caffeic acid, which is found in coffee, blueberries, and select vegetables, has been demonstrated to possess notable antioxidant properties and has exhibited anti-inflammatory effects [91]. Ferulic acid, which is prevalent in rice bran, oats, and fruits such as apples and oranges, is renowned for its capacity to neutralize free radicals and safeguard the skin from UV damage. Gallic acid, which is found in green tea, strawberries, and raspberries, has remarkable antimicrobial and antioxidant properties [92]. These properties underscore the importance of these compounds in protecting and promoting human health. In addition to contributing to the prevention of chronic diseases, these polyphenols play a crucial role in modulating the gut microbiota. This, in turn, influences the health of the central nervous system and the prevention of neurodegenerative diseases [93,94].

### 4.4. Other Relevant Microbial Metabolites

The gut microbiota produces a variety of other metabolites that also play a role in modulating brain function and neuroinflammation. Of particular interest among these are neuromodulators such as serotonin and dopamine, which are of central importance in regulating mood, cognition, and behavior. Neuromodulators are endogenous molecules, such as neurotransmitters and hormones, that regulate the activity of neurons by modifying the efficacy of synaptic signaling in the CNS [95]. 

Although primarily synthesized in the gut, serotonin exerts significant effects on the brain. Approximately 90% of the body’s serotonin is produced in the gut, with the microbiota serving as a pivotal contributor to this process. Serotonin is a principal neuromodulator involved in the regulation of mood, appetite, and sleep [96,97]. Studies have demonstrated that specific gut bacteria, including *Escherichia coli* and *Streptococcus*, can enhance serotonin synthesis in enterochromaffin cells within the gut [98]. This, in turn, can affect brain function through signaling along the vagus nerve. An imbalance in serotonin production, resulting from alterations in the microbiota, has been associated with disorders such as depression, anxiety, and irritable bowel syndrome, which exhibit neuropsychiatric components [99].

Dopamine, another key neurotransmitter, is produced in small amounts by the gut microbiota from tyrosine. Although the majority of dopamine is produced in the brain, a minor proportion is synthesized by the gut microbiota. Bacteria such as *Bacillus* and *Serratia* are capable of producing dopamine from precursors such as tyrosine. Dopamine plays a pivotal role in regulating motor functions, motivation, and reward [100]. Alterations in dopamine levels, which can be influenced by the composition of the microbiota, are implicated in neurodegenerative diseases such as PD, where the loss of dopamine in the brain leads to the characteristic motor symptoms [101].

## 5. Interaction Mechanisms between Microbial Metabolites and the CNS

The communication between the intestinal microbiota and the CNS is a complex and multifactorial process, which is carried out through several mechanisms. These include the regulation of physical barriers such as the intestinal barrier and the BBB, the modulation of inflammation and neuroinflammation, the influence on neurotransmission and synaptic plasticity, and the control of oxidative stress and neuronal apoptosis. These mechanisms allow microbial metabolites to play a fundamental role in neurological health and disease.

### 5.1. Intestinal Barrier and Blood–Brain Barrier

The maintenance of homeostasis between the gut and the brain is contingent upon the integrity of the intestinal permeability and the BBB. The gut microbiota and its metabolites play a pivotal role in regulating these barriers. SCFAs, such as butyrate, are vital for maintaining gut barrier integrity. This metabolite provides energy to colonocytes and promotes the expression of tight junction proteins, such as claudin and occludin, which are essential for preventing excessive gut barrier permeability. When the gut barrier is compromised, the translocation of bacteria and their components, such as LPS, into the systemic circulation increases, triggering inflammatory responses that can have effects throughout the body, including the CNS [51].

The BBB, which separates the brain from the systemic circulation, can also be affected by changes in intestinal permeability. Microbial products such as SCFAs can modulate the integrity of this barrier through the regulation of systemic inflammation. An increase in intestinal permeability, accompanied by the translocation of LPSs and other proinflammatory molecules, can induce BBB dysfunction, allowing the entry of potentially neurotoxic substances into the brain. This is especially relevant in neurodegenerative diseases, where the breakdown of the BBB can exacerbate neuroinflammation and the progression of the pathology. Thus, the intestinal microbiota and its metabolites not only modulate intestinal permeability, but also influence the protection and functionality of the CNS by affecting the BBB [102,103].

### 5.2. Activation of Inflammatory Pathways and Neuroinflammation

Systemic and neuroinflammatory processes are interrelated and play a pivotal role in the pathogenesis of neurodegenerative diseases. Microbial metabolites, including SCFAs, LPSs, and amino acid-derived products, have been demonstrated to act as key modulators of the immune response, both within the gut and in the brain. In particular, SCFAs, such as butyrate, have anti-inflammatory properties that are mediated through the activation of Treg cells, which suppress excessive inflammatory responses. This anti-inflammatory effect can reduce the production of proinflammatory cytokines which are key mediators of neuroinflammation [104,105,106].

In the CNS, the chronic activation of microglia and astrocytes, the primary immune cells of the brain, is a defining feature of neuroinflammation in neurodegenerative diseases. Intestinal dysbiosis and increased intestinal permeability can result in the systemic release of LPSs and other proinflammatory factors, which cross the blood–brain barrier and activate microglia. Once activated, microglia enter a proinflammatory state, releasing cytokines that perpetuate inflammation and neuronal damage. This sustained microglial activation not only contributes to direct neuronal death but may also interfere with neuronal repair and synaptic plasticity, exacerbating the progression of neurodegenerative diseases [107,108].

### 5.3. Role in Oxidative Stress and Neuronal Apoptosis

Oxidative stress represents a complex biological process whereby an imbalance occurs between the production of ROS and the body’s ability to neutralize them through its antioxidant systems. ROS encompass free radicals such as superoxide (O^2−^) and hydrogen peroxide (H_2_O_2_), which are byproducts of the cellular metabolism under normal conditions. However, when their production exceeds the body’s antioxidant capacity, these reactive species can damage crucial cellular components, including lipids, proteins, and DNA, thereby contributing to cellular dysfunction and premature cell death [109].

In the context of neurodegenerative diseases, such as AD, PD, and MS, oxidative stress has been identified as a central pathogenic factor. Neurons are particularly vulnerable to oxidative damage due to their high metabolic rate and limited regenerative capacity. The accumulation of oxidative damage over time can induce mitochondrial dysfunction, compromise the integrity of the blood–brain barrier, and activate signaling pathways leading to neuronal apoptosis, a type of programmed cell death that plays an important role in the progression of these diseases [110,111].

Microbial metabolites have the capacity to exert a direct influence on oxidative stress within the CNS. Some of these metabolites possess antioxidant properties, whereas others may act as pro-oxidants, thereby modulating the organism’s redox balance [112]. For instance, polyphenols, which are abundant in fruits, vegetables, and other plant-based foods, are metabolized by intestinal bacteria into active derivatives with antioxidant capacity. These polyphenolic metabolites can cross the BBB and act in the CNS, where they neutralize ROS, reducing oxidative damage and protecting neurons [113].

The antioxidant effect of polyphenol-derived metabolites is of great importance, as they not only prevent oxidative damage but also modulate cell signaling pathways that promote neuronal survival and neurogenesis [114]. By reducing oxidative stress, these metabolites assist in maintaining cellular homeostasis and preventing the activation of apoptotic cascades. Neuronal apoptosis is a process that is initiated when cell damage is irreversible. In the context of neurodegenerative diseases, this apoptosis contributes to the progressive loss of neurons and the cognitive and motor impairment associated with these diseases [115].

In addition to exerting antioxidant effects, some microbial metabolites have been observed to induce neuronal apoptosis in instances of pathological conditions. LPS can induce a systemic inflammatory response, which may increase ROS production in the brain [116]. This, in turn, may trigger signaling pathways that culminate in the activation of caspases, the enzymes responsible for the execution of apoptosis. When LPS enters the systemic circulation, it can cross the BBB, especially when this barrier is compromised, as occurs in many neurodegenerative diseases. Once in the brain, LPSs can trigger an inflammatory cascade that increases the production of ROS in glial cells, particularly in microglia [117,118].

An excess of ROS, when combined with proinflammatory cytokines such as TNF-α and IL-1β, can induce severe oxidative stress. This, in turn, activates intracellular signaling pathways that ultimately result in the activation of caspases. These enzymes are the primary mediators of apoptosis, resulting in DNA fragmentation, chromatin condensation, and, ultimately, programmed cell death of neurons [119]. This process of LPS- and ROS-induced apoptosis is of particular relevance in the pathogenesis of various neurodegenerative diseases. The gut microbiota also produces metabolites with neuroprotective effects that can counteract these aforementioned negative effects. One such metabolite is butyrate, which is mainly produced by the fermentation of dietary fibers by intestinal bacteria such as *Faecalibacterium prausnitzii* and *Roseburia* spp. [120,121].

**Figure 2:**
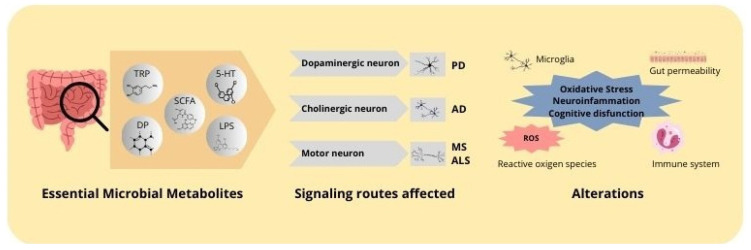
Mechanisms of action of microbial metabolites in neurodegenerative diseases. Microbial metabolites, including butyrate, lipopolysaccharides, neurotransmitters, and tryptophan, affect the progression of neurodegenerative diseases. These metabolites exert an impact on essential signaling pathways within the central nervous system, affecting dopaminergic, cholinergic, and motor neurons. The principal alterations include inflammation, oxidative stress, and mitochondrial dysfunction, which are associated with the pathogenesis of neurodegenerative diseases.

This equilibrium between microbial metabolites that stimulate apoptosis and those that inhibit it exemplifies the intricate interplay between the gut microbiota and the nervous system. As previously indicated, while certain metabolites, such as LPS, can exacerbate neuronal damage in pathological conditions, others, such as butyrate, exert neuroprotective effects, promoting neuronal survival and function. This duality indicates that the modulation of the gut microbiota and its metabolites may represent an effective therapeutic strategy for the prevention or treatment of neurodegenerative diseases. By shifting the balance towards a more neuroprotective and less neurotoxic environment in the CNS, modulation of the gut microbiota and its metabolites could potentially prevent or treat neurodegenerative diseases (Figure 2).

## 6. Empirical Evidence in Neurodegenerative Diseases

Recent research on the gut microbiota and its metabolites has demonstrated their substantial impact on a range of neurodegenerative disorders. These diseases, which include AD, PD, MS, ALS, and HD, present characteristic alterations in the gut microbiota that may contribute to the progression of neurodegeneration. This section presents an overview of the existing evidence on the relationship between changes in the microbiota, microbial metabolite profiles, and their impact on the pathogenesis of these diseases.

### 6.1. Alzheimer’s Disease

AD is typified by the progressive accumulation of extracellular beta-amyloid plaques and the intracellular formation of neurofibrillary tangles, which are composed of hyperphosphorylated tau proteins in brain tissue. These pathological deposits alter the brain architecture and contribute to a series of neurodegenerative processes, including synaptic dysfunction, the loss of neuronal connectivity, and, eventually, neuron death [122,123]. In recent years, there has been a growing interest in the potential role of the intestinal microbiota in the development of this pathology. A growing body of evidence indicates that individuals with AD frequently exhibit notable shifts in the composition of their intestinal microbiota. These alterations include an increase in proinflammatory bacteria and a reduction in those that produce beneficial metabolites, such as SCFAs. Such alterations may contribute to a systemic inflammatory state that exacerbates neuroinflammation, a key component in the progression of the disease [124].

Preclinical studies in animal models have demonstrated that gut dysbiosis can accelerate the deposition of beta-amyloid in the brain and exacerbate cognitive deficits. In this context, a study by Pishva et al. (2020) evaluated the influence of butyrate in a mouse model of induced AD. The administration of butyrate was observed to reduce the accumulation of amyloid plaques and improve cognitive function. This was achieved by decreasing neuroinflammation and modifying the expression of genes related to neurodegeneration [125]. A recent study (Winters and Vaughan, 2021) examined the role of quinolinic acid in AD. The findings indicated that this metabolite, derived from tryptophan, intensifies neuroinflammation. Animals that exhibited elevated levels of this metabolite demonstrated a considerable increase in microglia activation and astrogliosis, two indicators of brain inflammation. Furthermore, quinolinic acid facilitated excitotoxicity by enhancing glutamate release, which contributed to neurodegeneration. This study proposes that this metabolite may serve as a crucial mediator of AD progression, emphasizing its potential as a therapeutic target [126].

The findings of clinical studies in this domain have been substantiated by additional evidence. In 2019, Solvang and colleagues investigated the impact of the tryptophan pathway on cognitive performance in older adults. The results demonstrated that an imbalance in this metabolic pathway is associated with cognitive decline, indicating that proinflammatory metabolites may contribute to the pathogenesis of neurodegenerative diseases [127]. In 2020, Tanaka, Toldi, and Vécsei addressed the role of cytokines and bioactive tryptophan metabolites in neurodegenerative diseases. The results demonstrated that these metabolites exacerbate neuroinflammation and accelerate the progression of pathologies [128]. A study conducted in 2009 by Costantino addressed the role of the kynurenine pathway, the primary pathway for tryptophan degradation in neurodegenerative diseases. The study indicated that modulation of metabolites in this pathway can reduce neuroinflammation and, consequently, present therapeutic potential in neurodegenerative pathologies [129].

### 6.2. Parkinson’s Disease

PD is a neurodegenerative disorder that is characterized by the progressive loss of dopaminergic neurons in the substantia nigra. This leads to the development of hallmark motor symptoms, including rigidity, resting tremor, and bradykinesia. Additionally, patients may experience neuropsychiatric manifestations, such as depression and cognitive decline. In addition, patients with PD frequently exhibit gastrointestinal symptoms. Moreover, these patients frequently present with gastrointestinal symptoms, particularly constipation, which can precede the onset of motor symptoms by several years. This relationship has generated increasing interest within the scientific community regarding the potential involvement of the gut–brain axis in this pathophysiology. It has been suggested that alterations in the gut microbiota and gastrointestinal function may contribute to the initiation and progression of PD [130].

A growing body of evidence indicates that patients with PD exhibit intestinal dysbiosis, characterized by a reduction in SCFA-producing bacteria and an increase in proinflammatory species. This microbial imbalance may affect the production of metabolites that modulate inflammation both systemically and within the brain. As discussed in previous sections, the observed decrease in butyrate may contribute to the chronic activation of microglia, a phenomenon commonly observed in the brains of PD patients and closely associated with the progression of neurodegeneration. This persistent microglial activation is believed to exacerbate neuronal damage, further advancing the disease’s course [131,132].

Hatano et al. (2016) conducted a preclinical study utilizing metabolomic technologies to identify novel biomarkers for PD. The analysis of metabolite profiles in both patient samples and animal models revealed significant alterations in the uric acid pathway, suggesting a potential link between these changes, oxidative stress, and the neurodegenerative processes observed in this pathology [133]. Shao and Le (2019) also reviewed recent advances in metabolomic research, noting that preclinical studies have identified significant changes in metabolites, including lactate and SCFAs. These metabolites are of particular interest, as they may contribute to the mitochondrial dysfunction and neuroinflammation that are hallmarks of PD [134]. The study conducted by Chang et al. (2018) focused on alterations in the metabolic profile and kynurenine metabolism in the plasma of PD patients. Their results suggest that these metabolic changes may play a role in disease progression, potentially through the modulation of neuroinflammatory pathways [135].

Furthermore, the aggregation of alpha-synuclein, a protein that forms toxic aggregates in the neurons of patients with PD, has been increasingly linked to intestinal dysfunction. The latest research indicates that pathological alpha-synuclein may have its origin in the gut and subsequently propagate to the brain via the vagus nerve. This process may be influenced by the composition of the gut microbiota and the metabolites it produces. This proposed gut–brain connection in the pathogenesis of PD has opened new avenues for research, particularly with regard to the hypothesis that modulating the gut microbiota could delay or even prevent the onset of the disease. Clinical trials are currently investigating the use of probiotics and prebiotics to restore microbial balance in PD, with the objective of improving both motor and non-motor symptoms.

In this context, the study by Lv et al. (2022) investigated the impact of prolonged hyperglycemia on alpha-synuclein aggregation and dopaminergic neuronal loss in a mouse model of PD. The findings indicated that elevated glucose levels significantly contribute to the accumulation of alpha-synuclein in the brain, thereby exacerbating neurodegeneration in dopaminergic neurons. This indicates a vital correlation between metabolic derangement and the advancement of this pathology [136]. Earlier studies by Devi et al. (2008) and Stichel et al. (2007) also examined the role of alpha-synuclein in mitochondrial damage associated with PD. The research demonstrated that alpha-synuclein accumulates within mitochondria, where it disrupts complex I function, leading to increased oxidative stress and mitochondrial dysfunction. These alterations, including changes in mitochondrial morphology and impaired function, are critical factors contributing to the progression of neurodegeneration in PD [137,138].

### 6.3. Multiple Sclerosis

MS is a chronic autoimmune disorder of the CNS, defined by an immune-mediated inflammatory process that results in the demyelination of neuronal axons. The degradation of myelin, a lipid-rich sheath that insulates and protects nerve fibers, impairs the efficient conduction of electrical impulses along axons, resulting in disrupted communication between different regions of the brain, spinal cord, and peripheral nervous system. Individuals with MS present with a wide range of neurological symptoms, which vary in severity and may affect multiple functional domains. These symptoms include, but are not limited to, muscle weakness, spasticity, sensory disturbances, visual impairments, coordination and balance difficulties, as well as cognitive and psychiatric manifestations. The clinical heterogeneity observed in MS patients is reflective of the multifocal distribution of demyelinating lesions throughout the CNS, which contributes to the diverse manifestations of the disease. 

Preclinical investigations have demonstrated that modulating the microbiota, whether through dietary interventions or the administration of probiotics, can mitigate disease severity in MS animal models. At the clinical level, while research remains in its infancy, there is a growing interest in the use of microbial interventions to modulate disease progression. These approaches have the potential to complement existing immunomodulatory therapies, thereby providing an additional strategy for the management of neuroinflammation and the prevention of demyelination. Specifically, the study conducted by Nandakumar et al. (2018) investigated the role of the gut microbiota in an induced experimental autoimmune encephalomyelitis (EAE) model. The results indicated that modifications to the gut microbiota can impact both the onset and progression of the disease. In particular, alterations in the metabolic profile of gut-derived metabolites, such as SCFAs and other bioactive compounds, were observed to influence immune responses and central nervous system inflammation. These findings suggest that gut microbiota modulation may represent a promising therapeutic avenue for the management of MS [139].

In a further study, Bhargava and colleagues (2020) examined alterations in the bile acid metabolism among patients with MS and evaluated the potential of bile acid supplementation to attenuate neuroinflammation and improve clinical outcomes. The investigation revealed that the bile acid metabolism is disrupted in MS patients, with specific abnormalities in the levels of certain bile acids, indicating a possible imbalance in their metabolism. These findings indicate that bile acids may serve a role in modulating immune responses and mitigating inflammation within the CNS [140]. Consequently, bile acid modulation may represent a potential therapeutic strategy for managing MS by influencing the regulatory mechanisms of immune function and inflammatory processes.

A 2022 study by Ntranos et al. examined the presence and effects of bacterial neurotoxic metabolites in the cerebrospinal fluid (CSF) and plasma of patients with MS. The study identified the neurotoxic metabolites of bacterial origin in both CSF and plasma samples from MS patients. These metabolites, which include products of bacterial degradation and other potentially harmful compounds, may exert toxic effects on the CNS. This suggests that these bacterial neurotoxic metabolites could play a role in modulating neuroinflammation, thereby exacerbating the immune response and contributing to the neuronal damage characteristic of MS [141].

### 6.4. Other Neurodegenerative Diseases (ALS and Huntington’s Disease)

Emerging evidence indicates that the microbiota and its metabolites may be involved in the pathogenesis of other neurodegenerative diseases, including ALS and HD. Nevertheless, research in these domains remains relatively nascent and underdeveloped.

In ALS, a progressive neurodegenerative disease characterized by the degeneration of motor neurons and resultant paralysis, alterations in the composition of the intestinal microbiota have been observed. The latest research suggests that microbial metabolites may influence the progression of ALS by affecting inflammatory and immune responses. In particular, a reduction in SCFA-producing bacteria accompanied by an increase in proinflammatory bacterial species could create a neurotoxic environment that accelerates the degeneration of motor neurons. For example, lower levels of SCFAs and the proliferation of proinflammatory microbial taxa may exacerbate neuroinflammation and neuronal damage. 

The study conducted by Blacher et al. (2019) investigated the role of the gut microbiome and its metabolites in modulating ALS in murine models. The results demonstrated notable distinctions in the composition of the gut microbiome between ALS-induced mice and controls. In particular, alterations in the abundance of specific bacterial species were associated with disease progression. Furthermore, the study identified specific metabolites produced by the microbiome that influenced inflammation and neurodegeneration. Some of these bacterial metabolites exhibited beneficial effects by mitigating inflammation and reducing neuronal damage, suggesting a potential benefits for therapeutic intervention [142]. 

In HD, a neurodegenerative disorder caused by the expansion of CAG repeats in the HTT gene, the gut microbiota also appears to play a role in modulating the pathology. Although the disease is of genetic origin, the variability in disease progression observed suggests that environmental factors, such as the gut microbiota, may influence the severity of symptoms. Modulation of the microbiota has been demonstrated in models of HD to alter the expression of genes related to inflammation and cell death in the brain, thereby suggesting a potential link between microbial metabolites and disease progression.

In a study conducted by Rodrigues et al. (2021), the presence and levels of kynurenine pathway metabolites in the CSF and blood of patients with HD were investigated. The study identified significant alterations in the levels of several kynurenine pathway metabolites in both the CSF and blood of patients with HD compared to healthy controls. Notable changes were observed in metabolites such as kynurenic acid and various intermediate products. Furthermore, some of these metabolites exhibited a correlation with the severity of symptoms and disease progression. For instance, certain metabolites in the CSF were found to be associated with clinical markers of disease progression and the extent of motor dysfunction in HD patients [143]. 

## 7. Therapeutic Interventions and Gut Microbiota Modulation

The growing recognition of the gut microbiota influence on CNS health has accelerated research into innovative therapeutic strategies aimed at modulating this interaction. These approaches include the administration of prebiotics and probiotics, dietary interventions, FMT, and the development of pharmacological agents targeting microbial metabolites. The overarching objective of these strategies is to restore or optimize microbial balance and enhance the production of beneficial metabolites, with the ultimate aim of mitigating the progression of neurodegenerative diseases and improving patient quality of life.

### 7.1. Use of Prebiotics and Probiotics

Prebiotics and probiotics represent one of the most extensively researched strategies for influencing the composition of the gut microbiota and, consequently, the production of metabolites with neuroprotective effects. Prebiotics, such as non-digestible dietary fibers, serve as a substrate for microbial fermentation, thereby promoting the growth of beneficial bacteria that produce SCFAs and other metabolites with anti-inflammatory and neuroprotective properties [144]. Probiotics are defined as live microorganisms that, when administered in adequate amounts, are capable of the transient colonization of the intestine and the subsequent modification of the existing microbial community. A growing body of literature suggests that certain probiotics, such as strains of Lactobacillus and Bifidobacterium, are able to increase the production of SCFAs and other metabolites with positive effects on neurological health [145,146].

The number of clinical studies that have evaluated the efficacy of prebiotics and probiotics in patients with neurodegenerative diseases is still limited. However, the initial results are promising. For example, some trials have demonstrated that probiotic supplementation can improve the markers of systemic inflammation and reduce beta-amyloid burden in patients with AD [147]. In PD, probiotics have demonstrated benefits in improving gastrointestinal symptoms and modulating markers of inflammation. However, further research is required to assess their direct impact on disease progression. In animal models, prebiotics and probiotics have exhibited a notable capacity to influence neuroinflammation, neurogenesis, and synaptic function, which lends support to the notion that these interventions may have a therapeutic role in neurodegenerative diseases [148,149].

### 7.2. Diet and Its Impact on Microbiota and Metabolites

Dietary habits represent a primary modulator of the gut microbiota composition, consequently influencing the production of microbial metabolites. The influence of dietary patterns on CNS health is evidenced by their impact on the microbiota. The Mediterranean diet, which is rich in fruits, vegetables, whole grains, and healthy fats such as olive oil, has been identified as a dietary pattern with particularly beneficial effects on neurological health. This dietary pattern has been associated with a greater abundance of diverse microbiota, particularly those capable of producing SCFAs, which have been linked to anti-inflammatory and neuroprotective effects. Observational studies and clinical trials have demonstrated that adherence to the Mediterranean diet is associated with a reduced risk of developing AD and other neurodegenerative diseases, suggesting that the modulation of the microbiota through dietary modifications may represent an effective preventive strategy [150,151].

Another noteworthy dietary approach is the ketogenic diet, which is characterized by a high fat and low carbohydrate intake, leading to the induction of a state of ketosis. This dietary pattern has been employed for decades in the treatment of drug-resistant epilepsy, and recent studies indicate that it may confer benefits in other neurodegenerative diseases. The ketogenic diet alters the composition of the gut microbiota and increases the production of metabolites such as ketone bodies, which have neuroprotective and anti-inflammatory effects. In animal models of AD and MS, the ketogenic diet has been demonstrated to reduce neuroinflammation and improve cognitive and motor function, although evidence in humans is still limited [152].

### 7.3. Fecal Microbiota Transplantation (FMT)

FMT is a therapeutic intervention that involves the transfer of gut microbiota from a healthy donor to a recipient with the objective of restoring a balanced microbiome. Initially employed to treat recurrent Clostridium difficile infections, FMT has demonstrated promise in restoring the production of beneficial microbial metabolites in the context of neurodegenerative diseases. In animal models, FMT has been shown to be effective in reducing neuroinflammation and the accumulation of pathological proteins such as beta-amyloid and alpha-synuclein. These effects appear to be mediated by restoring the production of SCFAs and other neuroprotective metabolites.

While human studies are still underway, preliminary findings from several investigations indicate encouraging outcomes. In patients with PD, FMT has been demonstrated to alleviate both motor and non-motor symptoms, potentially through modulation of the microbiota and production of beneficial metabolites. However, the variability in response to this treatment suggests that a more comprehensive understanding of the underlying mechanisms and donor selection is necessary to optimize its efficacy and safety in the management of neurodegenerative diseases.

### 7.4. Microbial Metabolite-Based Drug Development

The development of pharmaceuticals based on microbial metabolites represents a novel and promising approach in the treatment of neurodegenerative disorders. The identification of specific microbial metabolites that influence neuroinflammation, neuroprotection, and synaptic plasticity provides novel avenues for therapeutic intervention. Among the most extensively studied metabolites are SCFAs, which have been demonstrated to exert direct effects on both systemic inflammation and brain health. Butyrate’s capacity to enhance BBB functionality and stimulate histone acetylation renders it a promising candidate for the development of therapies targeting neurodegenerative diseases [153,154].

Nevertheless, the transition of these preclinical findings into efficacious clinical treatments is beset with numerous challenges. One of the most significant challenges is the complexity and diversity of the human microbiome, which varies considerably between individuals and can influence the response to treatments based on microbial metabolites [155]. Furthermore, the stability and bioavailability of these metabolites in the human organism are crucial factors that must be addressed in drug development. Despite these challenges, advances in biotechnology and pharmacology are facilitating the development of compounds derived from microbial metabolites, with some already in early phases of clinical trials [156].

The modulation of the gut microbiota and its metabolites represents an innovative and promising therapeutic approach for the treatment of neurodegenerative diseases. As research progresses, these strategies are likely to be integrated into standard therapies, thereby providing new avenues for mitigating the progression of these diseases and improving the quality of life of patients.

## 8. Challenges and Future Perspectives

The study of the gut microbiota and its metabolites has led to the identification of new avenues for understanding and treating neurodegenerative diseases. However, this emerging field is confronted with a number of challenges and limitations that must be overcome in order to translate preclinical findings into clinically effective treatments.

### 8.1. Current Limitations in Research

The accurate identification and quantification of relevant microbial metabolites remains a significant challenge due to the intricate composition of the human microbiome and the limited sensitivity of current analytical techniques. The reliance on animal models introduces an additional layer of complexity, as differences in microbiota and physiology can influence the relevance of preclinical findings. Furthermore, the variability in experimental methods hinders the consistent application of results in a clinical context.

### 8.2. Emergent Areas

Notwithstanding these challenges, advances in metabolomics are enhancing the characterization of microbial metabolites in diverse biological matrices. Humanized animal models and brain organoids are emerging as more pertinent platforms for investigating the impact of the human microbiome on neurodegeneration. 

Moreover, integrating omics approaches (genomics, transcriptomics, and proteomics) with metabolomics may elucidate novel therapeutic targets and biomarkers for neurodegenerative diseases.

### 8.3. Personalized Medicine Perspective

**Figure 3:**
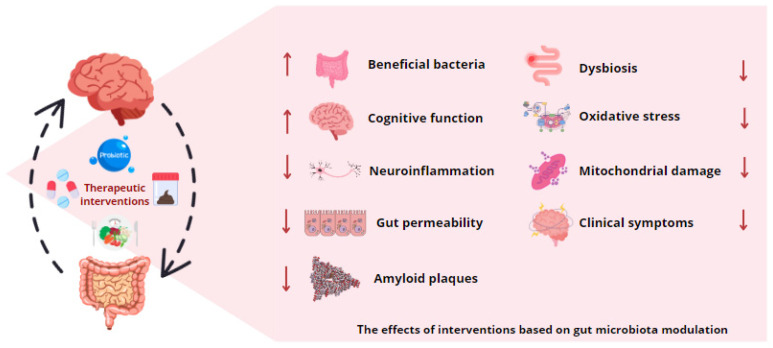
Gut microbiota modulation as therapy for neurodegenerative diseases. The application of various therapeutic interventions based on gut microbiota modification, including probiotics, prebiotics, dietary modifications, antibiotics, and fecal microbiota transplantation, has been observed to influence the production of microbial metabolites. These microbial byproducts have been demonstrated to possess the potential to mitigate intestinal dysbiosis, stimulate the proliferation of bacterial strains with neuroprotective and anti-inflammatory properties, and exert beneficial effects on the symptomatology associated with neurodegenerative diseases.

The use of personalized medicine based on microbial profiles represents a promising avenue for predicting the risk of neurodegenerative diseases and tailoring therapies. The analysis of individual microbial profiles may facilitate the forecasting of disease susceptibility and the guidance of early interventions. However, the implementation of such personalized therapies is confronted with a number of challenges, including the necessity for precise analytical technologies and a more profound comprehension of the impact of external factors on microbiota and metabolite production (Figure 3).

## 9. Conclusions

Research on the gut microbiota and its metabolites has yielded significant insights into the pathogenesis and potential treatment of neurodegenerative diseases. This review elucidates the function of microbial metabolites, including SCFAs, amino acids, and other bioactive compounds, in regulating neuroinflammation, synaptic function, and neuroprotection. These metabolites impact CNS health through diverse mechanisms, such as the modulation of intestinal permeability, immune responses, and neuronal signaling. Evidence from both preclinical and clinical studies correlates dysbiosis and altered metabolite production with diseases like AD, PD, and MS.

### 9.1. Practical Applications

A deeper comprehension of the microbiota’s influence on neurodegenerative disorders may facilitate the development of novel therapeutic strategies. Interventions such as prebiotics, probiotics, dietary modifications, and fecal microbiota transplantation have demonstrated potential for both treatment and prevention. Moreover, pharmacological agents derived from microbial metabolites may offer targeted therapies to decelerate disease progression. The identification of specific microbial profiles associated with CNS health may also provide biomarkers for early diagnosis and personalized treatment.

### 9.2. Future Lines of Research

The field of microbiota research continues to evolve, with numerous questions yet to be addressed. Current challenges include enhancing metabolite analysis techniques, translating preclinical findings to human applications, and accounting for inter-individual variability in microbiota modulation responses. Future research should prioritize the advancement of metabolomic technologies, the development of improved humanized animal models, and the implementation of rigorous clinical trials to assess the efficacy and safety of microbiota-based interventions.

## Data Availability

Not applicable.
